# Supplementary figures and images for: Differential insulin response characteristics of graphene oxide–gold nanoparticle composites under varied synthesis conditions
Source: PLoS One. 2025 Jan 13;20(1):e0317126. doi: 10.1371/journal.pone.0317126 (PMC11730386; doi:10.1371/journal.pone.0317126)

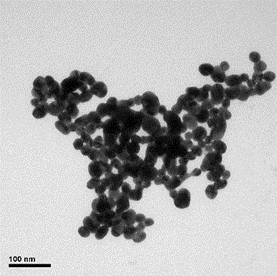

Supplement: S1 Fig — (TIF) [file pone.0317126.s001.tif]
