# Supplementary material for: Differential insulin response characteristics of graphene oxide–gold nanoparticle composites under varied synthesis conditions
Source: PLoS One. 2025 Jan 13;20(1):e0317126. doi: 10.1371/journal.pone.0317126 (PMC11730386; doi:10.1371/journal.pone.0317126)
Supplement: S1 Code — (DOCX) [file pone.0317126.s003.docx]

clc;clear;

path='E:\20221213';

A = dir(fullfile(path,'*.txt'));

A = struct2cell(A);

num = size(A);

x=A(1,:);

for k = 1:num(2)

newpath = strcat(path,'\',x(k));

temp=x(k);

temp=char(temp);

flength=length(temp);

dotnum=0;

for j=1:flength

if temp(j)=='.'

dotnum=j;

end

infname=temp(1:dotnum-1);

end

load(char(newpath));

data=load(char(newpath));

newpath2 = strcat(path,'\',infname);

save(newpath2,char(infname));

max1=data(54,2);

index1=54;

for i=54:608

if max1<data(i,2)

max1=data(i,2);

index1=i;

end

end

peak1(1,1)=data(index1,1);

peak1(1,2)=max1;

max2=data(750,2);

index2=750;

for i=750:1338

if max2<data(i,2)

max2=data(i,2);

index2=i;

end

end

peak2(1,1)=data(index2,1);

peak2(1,2)=max2;

end

%pure insulin

x=hyj1d5control(:,1);

figure;

plot(x,hyj1d5control(:,2));

hold on

plot(x,hyj1d5nyds01d5(:,2));

hold on

plot(x,hyj1d5nyds03(:,2));

hold on

plot(x,hyj1d5nyds06(:,2));

hold on

plot(x,hyj1d5nyds09(:,2));

hold on

plot(x,hyj1d5nyds12(:,2));

hold on

plot(x,hyj1d5nyds15(:,2));

hold on

plot(x,hyj1d5nyds18(:,2));

hold on

plot(x,hyj1d5nyds21(:,2));

hold on

plot(x,hyj1d5nyds24(:,2));

hold on

plot(x,hyj1d5nyds27(:,2));

hold on

plot(x,hyj1d5nyds30(:,2));

xlabel('Wavelength/nm');

ylabel('Abs');

legend('Control','1.5 μM','3 μM','6 μM','9 μM','12 μM','15 μM','18 μM','21 μM','24 μM','27 μM','30 μM')

%find the absorbance peak

max=hyj1d5control(894,2);

index1=894;

for i=894:1128

if max<hyj1d5control(i,2)

max=hyj1d5control(i,2);

index1=i;

end

end

peak_max(1,:)=[hyj1d5control(index1,1),max];

max3=hyj1d5control(54,2);

index3=54;

for i=54:607

if max3<hyj1d5control(i,2)

max3=hyj1d5control(i,2);

index3=i;

end

end

peak_max3(1,:)=[hyj1d5control(index3,1),max3];

Absrate3(1,:)=peak_max(1,2)/peak_max3(1,2);

max=hyj1d5nyds18(894,2);

index1=894;

for i=894:1128

if max<hyj1d5nyds18(i,2)

max=hyj1d5nyds18(i,2);

index1=i;

end

end

peak_max(2,:)=[hyj1d5nyds18(index1,1),max];

max3=hyj1d5nyds18(54,2);

index3=54;

for i=54:607

if max3<hyj1d5nyds18(i,2)

max3=hyj1d5nyds18(i,2);

index3=i;

end

end

peak_max3(2,:)=[hyj1d5nyds18(index3,1),max3];

Absrate3(2,:)=peak_max(2,2)/peak_max3(2,2);

max=hyj1d5nyds21(894,2);

index1=894;

for i=894:1128

if max<hyj1d5nyds21(i,2)

max=hyj1d5nyds21(i,2);

index1=i;

end

end

peak_max(3,:)=[hyj1d5nyds21(index1,1),max];

max3=hyj1d5nyds21(54,2);

index3=54;

for i=54:607

if max3<hyj1d5nyds21(i,2)

max3=hyj1d5nyds21(i,2);

index3=i;

end

end

peak_max3(3,:)=[hyj1d5nyds21(index3,1),max3];

Absrate3(3,:)=peak_max(3,2)/peak_max3(3,2);

max=hyj1d5nyds24(894,2);

index1=894;

for i=894:1128

if max<hyj1d5nyds24(i,2)

max=hyj1d5nyds24(i,2);

index1=i;

end

end

peak_max(4,:)=[hyj1d5nyds24(index1,1),max];

max3=hyj1d5nyds24(54,2);

index3=54;

for i=54:607

if max3<hyj1d5nyds24(i,2)

max3=hyj1d5nyds24(i,2);

index3=i;

end

end

peak_max3(4,:)=[hyj1d5nyds24(index3,1),max3];

Absrate3(4,:)=peak_max(4,2)/peak_max3(4,2);

max=hyj1d5nyds27(894,2);

index1=894;

for i=894:1128

if max<hyj1d5nyds27(i,2)

max=hyj1d5nyds27(i,2);

index1=i;

end

end

peak_max(5,:)=[hyj1d5nyds27(index1,1),max];

max3=hyj1d5nyds27(54,2);

index3=54;

for i=54:607

if max3<hyj1d5nyds27(i,2)

max3=hyj1d5nyds27(i,2);

index3=i;

end

end

peak_max3(5,:)=[hyj1d5nyds27(index3,1),max3];

Absrate3(5,:)=peak_max(5,2)/peak_max3(5,2);

max=hyj1d5nyds30(894,2);

index1=894;

for i=894:1128

if max<hyj1d5nyds30(i,2)

max=hyj1d5nyds30(i,2);

index1=i;

end

end

peak_max(6,:)=[hyj1d5nyds30(index1,1),max];

max3=hyj1d5nyds30(54,2);

index3=54;

for i=54:607

if max3<hyj1d5nyds30(i,2)

max3=hyj1d5nyds30(i,2);

index3=i;

end

end

peak_max3(6,:)=[hyj1d5nyds30(index3,1),max3];

Absrate3(6,:)=peak_max(6,2)/peak_max3(6,2);

%commercial insulin

x=hyj1d5control_1(:,1);

figure;

plot(x,hyj1d5control_1(:,2));

hold on

plot(x,hyj1d5cz05_1(:,2));

hold on

plot(x,hyj1d5cz10_1(:,2));

hold on

plot(x,hyj1d5cz15_1(:,2));

hold on

plot(x,hyj1d5cz20_1(:,2));

hold on

plot(x,hyj1d5cz25_1(:,2));

xlabel('Wavelength/nm');

ylabel('Abs');

legend('Control','5 μM','10 μM','15 μM','20 μM','25 μM')

%find the absorbance peak

max=hyj1d5control_1(894,2);

index1=894;

for i=894:1128

if max<hyj1d5control_1(i,2)

max=hyj1d5control_1(i,2);

index1=i;

end

end

peak_max(1,:)=[hyj1d5control_1(index1,1),max];

max=hyj1d5cz05_1(894,2);

index1=894;

for i=894:1128

if max<hyj1d5cz05_1(i,2)

max=hyj1d5cz05_1(i,2);

index1=i;

end

end

peak_max(2,:)=[hyj1d5cz05_1(index1,1),max];

max=hyj1d5cz10_1(894,2);

index1=894;

for i=894:1128

if max<hyj1d5cz10_1(i,2)

max=hyj1d5cz10_1(i,2);

index1=i;

end

end

peak_max(3,:)=[hyj1d5cz10_1(index1,1),max];

max=hyj1d5cz15_1(894,2);

index1=894;

for i=894:1128

if max<hyj1d5cz15_1(i,2)

max=hyj1d5cz15_1(i,2);

index1=i;

end

end

peak_max(4,:)=[hyj1d5cz15_1(index1,1),max];

max=hyj1d5cz20_1(894,2);

index1=894;

for i=894:1128

if max<hyj1d5cz20_1(i,2)

max=hyj1d5cz20_1(i,2);

index1=i;

end

end

peak_max(5,:)=[hyj1d5cz20_1(index1,1),max];

max=hyj1d5cz25_1(894,2);

index1=894;

for i=894:1128

if max<hyj1d5cz25_1(i,2)

max=hyj1d5cz25_1(i,2);

index1=i;

end

end

peak_max(6,:)=[hyj1d5cz25_1(index1,1),max];
