# Supplementary material for: Differential insulin response characteristics of graphene oxide–gold nanoparticle composites under varied synthesis conditions
Source: PLoS One. 2025 Jan 13;20(1):e0317126. doi: 10.1371/journal.pone.0317126 (PMC11730386; doi:10.1371/journal.pone.0317126)
Supplement: Attachment — Submitted filename: Reviewer Comments.docx [file pone.0317126.s004.docx]

Manuscript number – PONE-D-24-28170

Differential insulin response characteristics of graphene oxide–gold nanoparticle composites under varied synthesis conditions

Qian Zhang^1^, Yanjun Pan^1^, Jin Pan^1^, Zhichen Wang^1^, Ruyi Lu^1^, Jing Sun^1^, Jingyi Feng^1^*

The authors has worked on Pure and commercial Insulin detection by the help of AuNPs/GO and also examined its response characteristics to the representative products of the five commercial Insulin categories and demonstrated the ability to accurately determine unknown concentrations of commercial insulin, confirming their potential applications in drug sensing. This research group has wide experience on development of biological activities.

1) Page number – 10 Line number - 25

It is mentioned GO/GNPs in full article. It should be AuNPs/GO in place of GO/GNPs.

2) Page number – 16 Line number - 145

The full form of instruments are mentioned with the abbreviation but in this case of EIS it is not mentioned.

3) The Chloroauric acid is costly so how should author justify the cost-effectiveness of the synthesized product.

I recommend this article to accept after minor revision.
